# Light trapping and guided mode enhancement in perovskite/Si tandem solar cells with embedded silicon nanowires

**DOI:** 10.1039/d5ra07413d

**Published:** 2025-12-09

**Authors:** Arpan Saha, Mohammad Ajmain Fatin, Tasmiat Rahman, Mainul Hossain

**Affiliations:** a Department of Electrical and Electronic Engineering, University of Dhaka Dhaka-1000 Bangladesh mainul.eee@du.ac.bd; b Department of Electronics and Computer Science, University of Southampton Southampton SO17 1BJ UK

## Abstract

Energy loss at the interface between the subcells limits the efficiency of existing tandem solar cells. In this work, we propose a novel two-terminal perovskite/silicon tandem solar cell with vertically aligned silicon (Si) nanowires (NWs) incorporated between the two subcells. The sub-wavelength dimensions of the embedded Si NWs, grown on top of the Si bottom subcell, allow efficient light trapping and lead to guided resonant modes. Finite-difference time-domain (FDTD) analysis shows that, across the entire spectrum, these guided modes effectively couple the incident light between the subcells, reducing reflection losses in the interlayer and enhancing absorption in the underlying bottom c-Si cell. To evaluate the performance of the proposed tandem solar cell, we performed electrical simulations using the enhanced carrier generation profile obtained from the FDTD simulations. The proposed embedded NW tandem configuration yields 22.6% enhancement in short-circuit current density compared to conventional architectures, boosting the power conversion efficiency from 26.98% to 32.11%. These findings offer the necessary theoretical framework for experimentalists, providing a clear pathway towards realizing high-performance perovskite/Si tandem solar cells.

## Abstract 2

Embedding Si nanowires between the perovskite and silicon subcells enables strong light-trapping and guided-mode coupling, boosting current generation and raising tandem efficiency from 26.98% to 32.11%.

## Graphical abstract

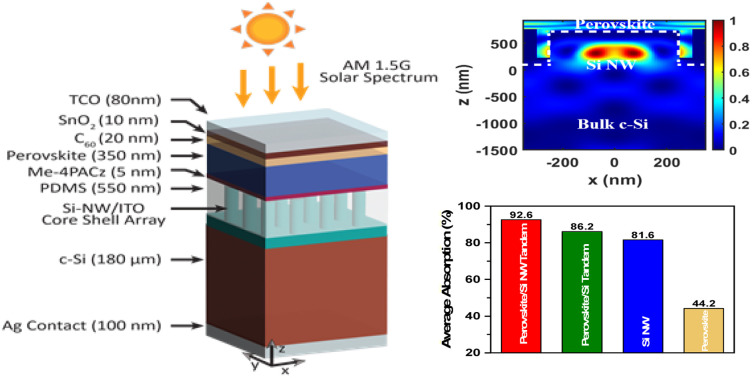

## 1. Introduction

Crystalline silicon (c-Si) has dominated the photovoltaics (PV) market for an extensive period due to well-established processes that yield low-cost and highly stable solar cells with excellent power conversion efficiencies (PCEs). In recent years, perovskite solar cells have attracted a great deal of attention as a promising alternative to c-Si, due to their tunable bandgaps, high absorption coefficients, simpler manufacturing processes, higher PCEs and longer electron and hole diffusion lengths to minimize recombination losses.^1,2^ However, the performance of both single junction perovskite and Si cells are limited by the Shockley–Queisser (S–Q) limit.^3^ To surpass the S–Q limit, a standalone wide bandgap top perovskite solar cell is stacked with a standalone bottom Si solar cell having a narrower bandgap to form a tandem solar cell. The high energy photons from the incident sunlight are absorbed by the top subcell while the bottom subcell absorbs the low energy photons that pass through the top subcell. This allows absorption over a wider spectral range and hence boosts the PCE of the tandem solar cell.^4,5^ Despite the merits, reflection and recombination losses and poor light trapping at the interface between the two subcells degrade tandem solar cell performance. Some attempts to reduce the interlayer losses include the use of spectrally selective interlayers in the perovskite/Si tandems^6^ and the mechanical bonding of the subcells through a low-index epoxy as in III–V tandems.^7^ Monolithic 2-terminal perovskite/Si tandem solar cells have benefited from the integration of sub-micrometer scale sinusoidal nanotextures at the front surface of the Si subcell, achieving certified PCE as high as 29.8%.^8^ For instance, growing III–V nanowires (NWs) on top of Si substrates have enabled multi-junction solar cells with high efficiency when the subcell currents are matched.^8,9^ The subwavelength dimensions and high refractive indices of the NWs offer highly efficient broadband light absorption through an optical antenna effect.^10^ Finite-difference time-domain (FDTD) analysis confirmed that the one-dimensional (1D) NWs enhance absorption by not only coupling the incident light into guided modes but also by efficiently transporting the sub-bandgap photons to the Si bottom cell.^11^ However, Si NWs suffer from indirect bandgap and low absorption coefficient of Si in the 600–1100 nm spectral range. The absorption and conversion efficiency in the visible range can be significantly improved by introducing perovskites, with a direct bandgap and a large absorption coefficient, into the Si NW array.^12^ Recent studies have reported the integration of perovskites with Si NW arrays for enhanced broadband absorption in hybrid photodetectors and solar cells. Asuo *et al.* successfully fabricated a hybrid photodetector that combined halide perovskite with vertically aligned Si NW arrays, exhibiting strong broadband absorption for wavelengths ranging from near ultraviolet to near-infrared.^13^ Yan *et al.* used technology computer-aided design (TCAD) simulations to evaluate the performance of a Si NW array/perovskite structure. The incorporation of a halide perovskite among the intrinsic regions of a vertically aligned Si axial p–i–n NW array significantly improved the performance of the resulting solar cell, absorbing light between 300–800 nm wavelengths and reaching a PCE as high as 13.3%.^12^

In this paper, we propose a two-terminal (2-T) perovskite/Si tandem solar cell where an array of vertically aligned Si NWs is grown on top of a crystalline-Si (c-Si) bottom subcell. A 50 nm thick layer of indium tin oxide (ITO) is deposited on top of Si NWs, followed by encapsulation with polydimethylsiloxane (PDMS). The height, period, diameter and geometry of the Si NWs can be optimized to maximize absorption in the bottom Si subcell. To demonstrate efficient light coupling by the NWs, FDTD simulations were carried out to investigate the power absorption profile and electric field pattern in the underlying bulk Si. Finally, the output characteristics of the proposed tandem solar cell is benchmarked against a conventional tandem architecture.

## 2. Device structure and simulation methodology

**Fig. 1:**
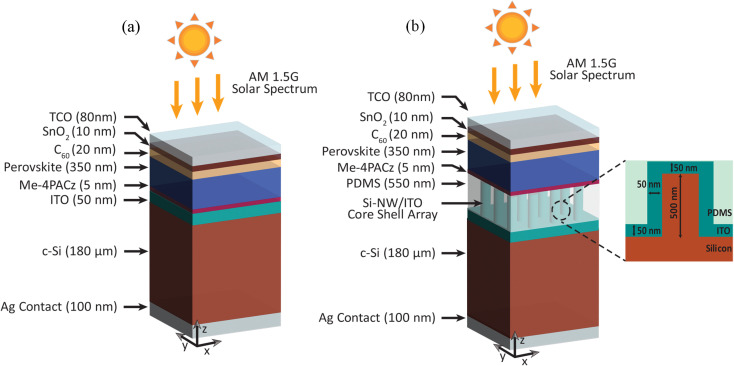
3D schematic of (a) planar two-terminal perovskite/Si tandem solar cell (b) proposed two-terminal perovskite/Si NW tandem solar cell with Si NW array embedded in the interlayer between the top and the bottom subcells. An enlarged version of a single Si NW surrounded by ITO and PDMS is shown with dimensions optimized for maximum absorption.

Fig. 1(a) shows the three-dimensional (3D) schematic of a conventional perovskite/Si tandem solar cell. The proposed device, shown in Fig. 1(b), consists of Si NWs in the interlayer between the two subcells. In each case, the tandem cell is irradiated from the top with AM1.5G, 1 sun spectrum. The vertically aligned Si NW/ITO core shells are grown on top of the 180 µm thick bulk c-Si, which serves as the bottom subcell of the tandem. A layer of PDMS is deposited around the Si NW/ITO core shell array. The ITO layer serves as the recombination junction between the perovskite and the c-Si subcells, confining all electronic transport and recombination beneath it. Since PDMS is deposited only after the ITO junction and lies entirely outside the electronically active layers, it does not affect vertical carrier transport or recombination. Its low refractive index is not detrimental, as PDMS is highly transparent and contributes negligible parasitic absorption; optical behavior is instead governed by the Si-NW/ITO geometry.^14^ PDMS primarily provides mechanical stabilization and environmental protection. High-aspect-ratio Si NWs are susceptible to bending or collapse during processing, and PDMS is well established as a conformal, transparent encapsulant that supports NW structures while preserving their optical performance. For instance, Fedorov *et al.*^15^ demonstrated gallium phosphide (GaP) NWs embedded within a PDMS membrane, where PDMS serves as a transparent, flexible, and chemically robust framework that preserves the NW morphology and protects the structures during optical operation. For the top perovskite subcell, an experimentally validated n–i–p wide-bandgap perovskite configuration is adopted. Cesium formamidinium lead iodide bromide [Cs_0.18_FA_0.82_Pb(I,Br)_3_] is chosen as the main absorber layer. This mixed halide perovskite has an optical bandgap of ∼1.70 eV which ensures strong visible-light absorption and appropriate current matching with the bottom c-Si sub cell. Furthermore, this wide-bandgap composition is also known to exhibit enhanced phase stability and reduced halide segregation under illumination, making it suitable for reliable tandem operation.^16^

The choice of carrier transport layers is also paramount for perovskite solar cell performance. Consequently, significant research efforts are being dedicated to designing robust, cost-effective, and efficient electron and hole transport layers (ETLs and HTLs). A major focus has been replacing the conventional but unstable and expensive HTL, spiro-OMeTAD. Promising alternative strategies include the development of inorganic nanoparticles,^17,18^ ionic liquids,^19^ dopant-free small molecules,^20^ and engineered metallophthalocyanines.^21^ These next-generation materials aim to provide superior durability and cost-effectiveness while enhancing both power conversion efficiency and long-term stability against environmental stressors like moisture and heat. Here, [4-(3,6-dimethyl-9*H*-carbazol-9-yl)butyl]phosphonic acid (Me-4PACz)—a self-assembled monolayer—is employed as the HTL. Me-4PACz is widely recognized for providing excellent band alignment, effective interfacial passivation, and efficient hole extraction in high-performance perovskite devices. Electron transport is facilitated by a C_60_/SnO_2_ bilayer ETL, where C_60_ acts as the primary electron-transport layer and the SnO_2_ overlayer forms a transparent, low-loss electron-selective contact that suppresses interfacial recombination and improves charge-collection stability. This C_60_/SnO_2_/Cs_0.18_FA_0.82_Pb(I,Br)_3_/Me-4PACz stack reflects the transport-layer architecture used in experimentally demonstrated wide-bandgap perovskite/Si tandem solar cells, ensuring that our simulations correspond to a practical and fabrication-feasible device configuration.^16,22^

To validate the simulation framework, the electrical performance of the standalone top and the bottom subcells was simulated using one-dimensional solar cell capacitance simulator (SCAPS-1D), which solves the coupled Poisson, transport, and continuity equations.^23^ All simulations were conducted at 300 K. The simulation results were benchmarked against experimentally reported device data under standard AM1.5G, 1-sun illumination.^16^ The simulated current density–voltage (*J*–*V*) curves (Fig. S1, SI) of the subcells exhibit close agreement with the corresponding experimental results (Table S1, SI), indicating that the optical and electrical models are accurately represented. This validation confirms that the calibrated subcells can be reliably used as the foundation for modeling the complete tandem device.

The optical simulations in this study were carried out using the finite-difference time-domain (FDTD) method which computes the electric and magnetic fields throughout the tandem solar cell by solving Maxwell's equations at every point in space and time. *A* plane wave source, with wavelengths ranging from *λ* = 300 nm to 1200 nm, illuminates the tandem cell from the top at normal incidence. The two-dimensional (2D) periodic structure of the Si NWs is in a square array and has a fourfold rotational symmetry about the propagation axis of light. Hence, both the transverse-electric (TE) and the transverse-magnetic (TM) polarizations yield the same absorption spectra when illuminated at normal incidence.^24^ Although polarization-dependent effects may emerge at different incident angles, a detailed angle-resolved analysis requires extensive optical modeling which lies outside the scope of the present study. Therefore, all FDTD simulations, in this work, are carried out with the TM waves alone, setting all polarization angles to 0°. Periodic boundary conditions were applied in the *x* and *y* directions which allows the periodic array to be modeled by carrying out simulations within the unit cell. Any reflected and transmitted fields were absorbed by applying perfectly matched layer (PML) boundary conditions on the top and the bottom surfaces along the *z* direction. A frequency domain monitor is placed at the top of the cell to calculate the reflectance *R*(*λ*) of the simulated structure. The absorbance, *A*(*λ*), of the tandem was calculated using the equation:

1 *A*(*λ*) = 1 − *R*(*λ*)

assuming that no light is transmitted once it passes the semi-infinite silicon bulk layer.

The two-dimensional (2D) frequency domain field and power monitors, placed within the unit cell, are used to compute the electric field and power absorption profiles. The absorbed power (*P*_abs_) per unit volume, electric field (*E*) and the permittivity (*ε*) of the medium, are all functions of position (*r*) and wavelength (*λ*) and can be expressed as:^25^2
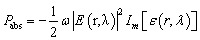
*P*_abs_ is normalized by dividing by the incident power at each wavelength. Assuming every photon absorbed yields one electron–hole pair, the carrier generation rate per unit volume (*G*(r)) can be expressed as:^25^3
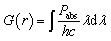
where *h* is Planck's constant and *c* is the speed of light in vacuum.

The dimensions of the Si NWs in the interlayer were optimized to maximize light absorption across the target spectral range. This is achieved by evaluating the absorption spectra obtained from the FDTD simulations at different NW radius (*r*_nw_) and period (*P*) while keeping the NW height fixed at 500 nm. Results show that the *r*_nw_ = 250 nm and *P* = 700 nm (Fig. S2, SI) yield the maximum absorption. Although increasing NW height can further strengthen light trapping and mode confinement, excessively tall NWs often suffer from mechanical instability caused by capillary forces during wet processing or stress arising from subsequent layer deposition.^23,24^ For this reason, the NW height was limited to 500 nm—chosen as a practical cutoff that balances optical enhancement with structural robustness. Fig. 1(b) shows an enlarged version of a single Si NW, surrounded by ITO and PDMS, with dimensions optimized for maximum absorption.

Finally, the optical generation profile from FDTD simulations was incorporated in SCAPS-1D to evaluate the current–voltage characteristics of the tandem solar cell. Device efficiency was further optimized through a current-matching approach by adjusting absorber thicknesses to match the subcell currents. All input parameters, including the interface defect densities, used in the electrical simulations were taken from well-established literature sources reporting standard and experimentally validated values (Tables S2 and S3, SI).

## 3. Proposed fabrication scheme

**Fig. 2:**
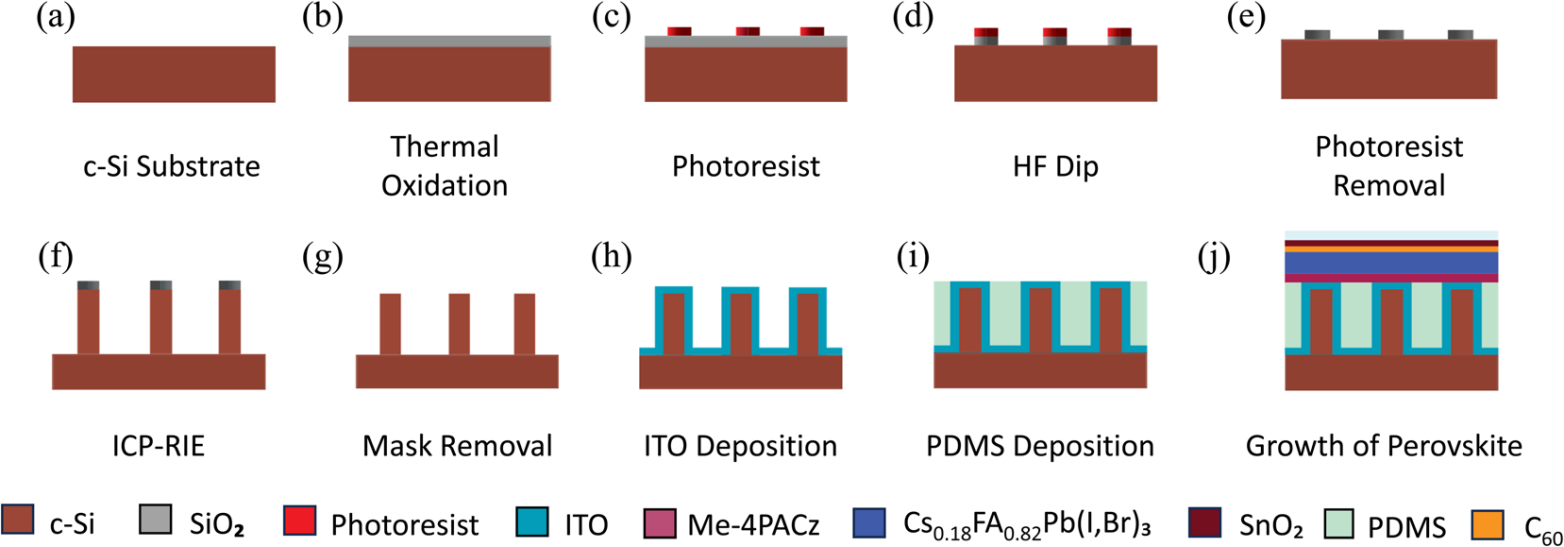
Fabrication scheme of the proposed perovskite/Si solar cell with vertically aligned Si NWs grown on top of the c-Si bottom cell. (a) c-Si substrate (b) wet thermal oxidation SiO_2_ (c) patterning using standard photolithography process (d) dipping into hydrofluoric acid (HF) solution to transfer the pattern (e) removal of photoresist using acetone (f) cryogenic dry etching of the patterned Si with inductively coupled plasma reactive ion etching (ICP-RIE) process to form the vertically aligned Si NWs (g) removal of photoresist masks (h) deposition of indium tin oxide (ITO) core shell using MOCVD (i) spin-coating of PDMS layer (j) deposition of HTL, perovskite and ETL.

Several methods have been adopted in the past to fabricate vertically aligned Si NW arrays. These include bottom-up techniques like vapor–liquid-solid (VLS)^26^ and supercritical fluid–liquid–solid (SFLS)^27^ mechanisms as well as more controllable top-down approaches like the metal-assisted chemical etching (MACE).^28^ The Si NWs proposed in our study can be fabricated using the combination of cryogenic inductively coupled plasma reactive ion etching (ICP-RIE) and photolithography as shown in Fig. 2. This method, demonstrated by Refino *et al.*^29^ yields doping independent, high aspect-ratio, vertically aligned Si NWs with smooth side walls and a homogeneous geometry. The c-Si substrate can be patterned using photolithography and then etched using plasma containing ions and radicals. Once the NWs are formed, a 50 nm thin layer of indium tin-oxide (ITO) can be deposited on top by horizontal-type MOCVD forming a core–shell structure. Pal *et al.* reported a similar In *P*–SiNW core–shell structure using MOCVD at atmospheric pressure.^30^ After the deposition of ITO, a PDMS layer can be deposited on top of the ITO-coated Si-NW array using a two-step spin coating process. In the first step, the PDMS uniformly fills up the dense Si-NW array. The excess residue of the PDMS on top of the array can be removed in the second step of the spin coating process which gives it a uniform planar configuration.^31^ After depositing the ITO shell, a Me-4PACz self-assembled monolayer is first spin-coated onto the ITO-coated Si NWs to form the hole-selective contact. The perovskite absorber is then deposited using a one-step spin-coating method followed by thermal annealing. A compact C_60_ electron-transport layer is subsequently added *via* thermal evaporation, and finally, a conformal SnO_2_ layer is deposited using low-temperature ALD to complete the device stack.^16^

## 4. Results and discussion

**Fig. 3:**
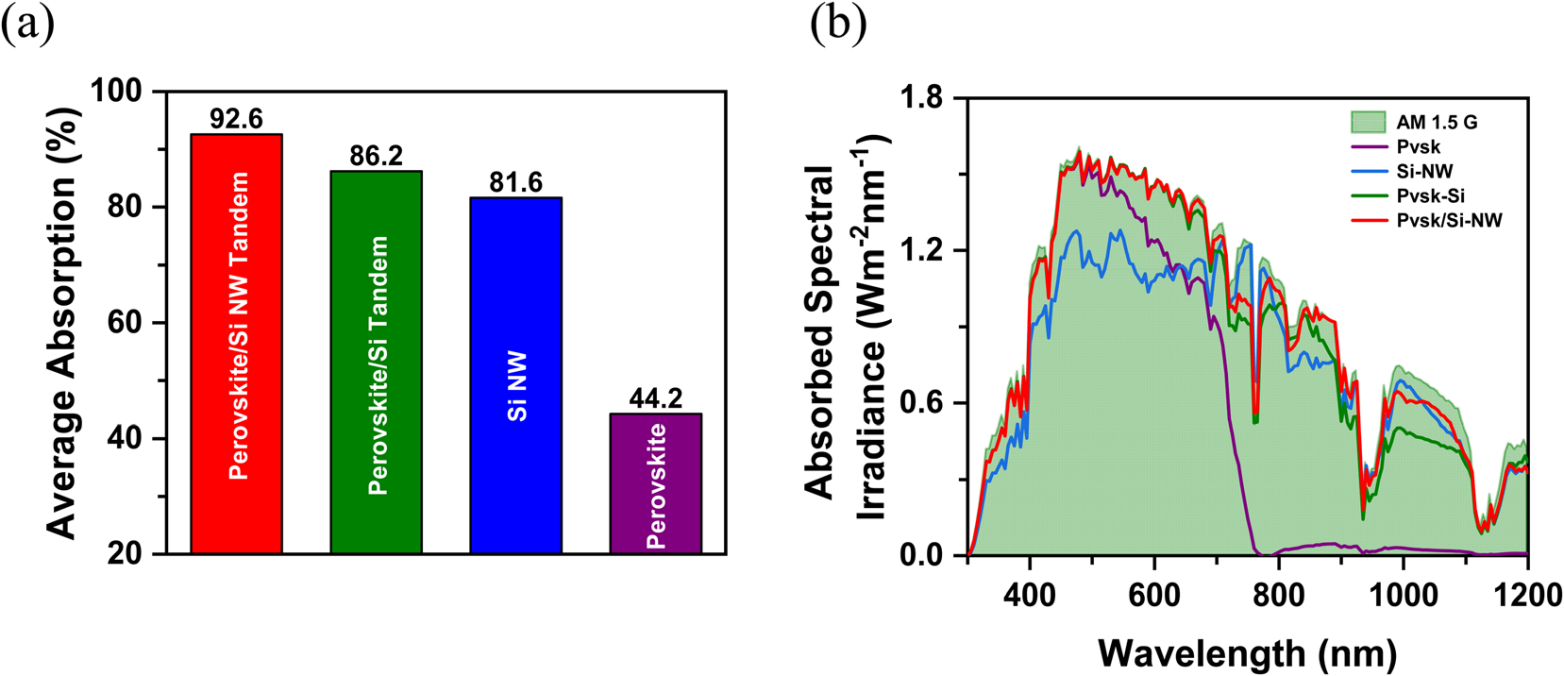
(a) Average absorption and (b) absorbed spectral irradiance under AM 1.5G of a standalone perovskite, a standalone Si NW array, a planar perovskite/Si tandem and a perovskite/Si NW solar cell over the entire spectral range.

Fig. 3(a) compares the average absorption (*A*_avg_) between the standalone perovskite solar cell (top subcell), a standalone c-Si solar cell with Si NW array (bottom subcell), a conventional perovskite/Si tandem solar cell and the proposed perovskite/Si NW solar cell with vertically aligned Si NWs grown on top of the bulk Si. The average absorption is defined as:^32^4
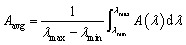


The corresponding absorbed spectral irradiance under AM 1.5G for the different solar cells are illustrated in Fig. 3(b). Compared to conventional perovskite/Si tandem solar cells, the proposed tandem design, with vertically aligned Si NWs in the interlayer, clearly shows enhanced absorption of the AM 1.5G spectrum, leading to an average absorption, *A*_avg_, as high as 92.6%. A standalone c-Si NW array exhibits superior absorption enhancement in the near infrared (NIR) region. Therefore, the integration of a top perovskite absorber layer with the light trapping and scattering capabilities of the Si NW array results in a highly efficient broadband absorber, outperforming other examined structures. The results are consistent with the previous study by Garnett *et al.* which demonstrated that ordered arrays of Si NWs can increase the path length of incident light by up to a factor of ∼73, enabling superior light-trapping and increased absorption over the entire AM1.5 G spectrum.^33^ The strong interaction between the incident light and the sub-wavelength Si NWs gives rise to a variety of optical effects that include wave-guiding, Fabry–Pérot resonances, low reflectivity (moth-eye effect), diffractive effects and near-field coupling.^34^ These effects are highly tunable by adjusting the NW array geometry and provide precise control on the reflection, absorption, and scattering properties of light for enhanced absorption.

### 4.1 Waveguiding effect

The vertically aligned Si NWs can act as subwavelength dielectric cylindrical waveguides that can efficiently couple incident light, parallel to its long axis, into leaky resonant modes. Earlier, simulations by Wang *et al.*^35^ have shown that the incident waves can only couple to HE_1*m*_ leaky modes, where 1 is the azimuthal mode number and *m* the radial mode number, which represents the radial variation of the electromagnetic field. The strength of the coupled modes depends on the wavelength of the incident light and can be tuned across the entire spectrum by simply varying the radius of the NW.

**Fig. 4:**
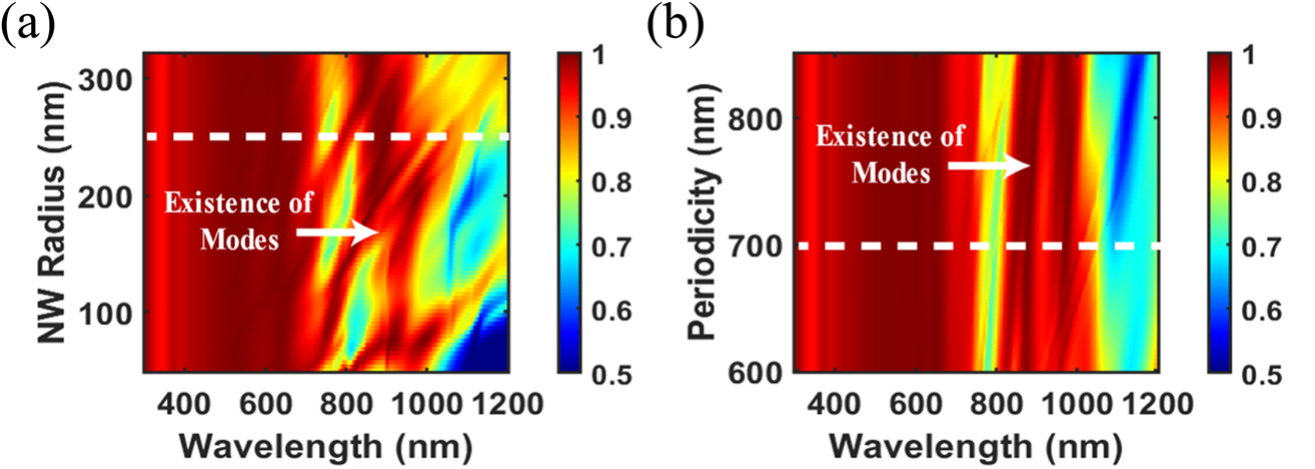
Contour plot of absorption as a function of the wavelength of incident light and (a) NW radius, (b) NW period in a perovskite/Si NW tandem. The dash-dotted white line represents NW with *r*_NW_ = 250 nm and *P* = 700 nm. The NW height is fixed at 500 nm.

Fig. 4(a) shows a contour plot of the simulated absorption characteristics of the proposed tandem as a function of wavelength of incident light and the NW radius. The colored bands correspond to guided mode resonances, where incident light is efficiently coupled into the NW array. The transverse leaky mode resonances, associated with the fundamental HE_11_ mode, are responsible for the broader red bands, where partial confinement of light enhances the field intensity before radiating into free space. With the increasing NW radii, the resonant modes are red shifted towards longer wavelengths. Also, NWs with larger radii (*r*_NW_) can sustain multiple modes. Due to the symmetric nature of the NWs and their longer dimensions, the interactions with the higher guided modes, and consequently the absorption enhancements, are much weaker. Hence, only the coupling of the fundamental HE_11_ mode primarily contributes to the absorption enhancement. For *r*_NW_ > 250 nm, longitudinal resonant modes appear for *λ* > 800 nm. These are weaker than the transverse modes, but can couple to them, giving rise to interference between the modal fields at longer wavelengths. Fig. 4(b) shows the variation of wavelength-dependent absorption characteristics with NW period. The white dashed line indicates the NW radius (Fig. 4(a)) and period (Fig. 4(b)) selected for the proposed structure. The chosen radius and period support multiple guided and leaky mode resonances across visible and near-infrared regions, ensuring efficient coupling of incident light into the underlying Si and is responsible for the enhanced broadband absorption.^11,34–36^ The NW array leverages the entire spectrum of incident light without requiring additional light-trapping techniques such as surface texturing. Moreover, the unique geometry of Si NWs permit increased optical path lengths and reduced reflections at the perovskite/Si interface.^37,38^ The scattering and coupling of light between adjacent Si NWs increase the absorption further by causing multiple interactions with the Si bottom cell. Also, the NWs restrict a sharp change in refractive index by providing gradual transition in the refractive index, leading to reduced reflection losses.^39^

**Fig. 5:**
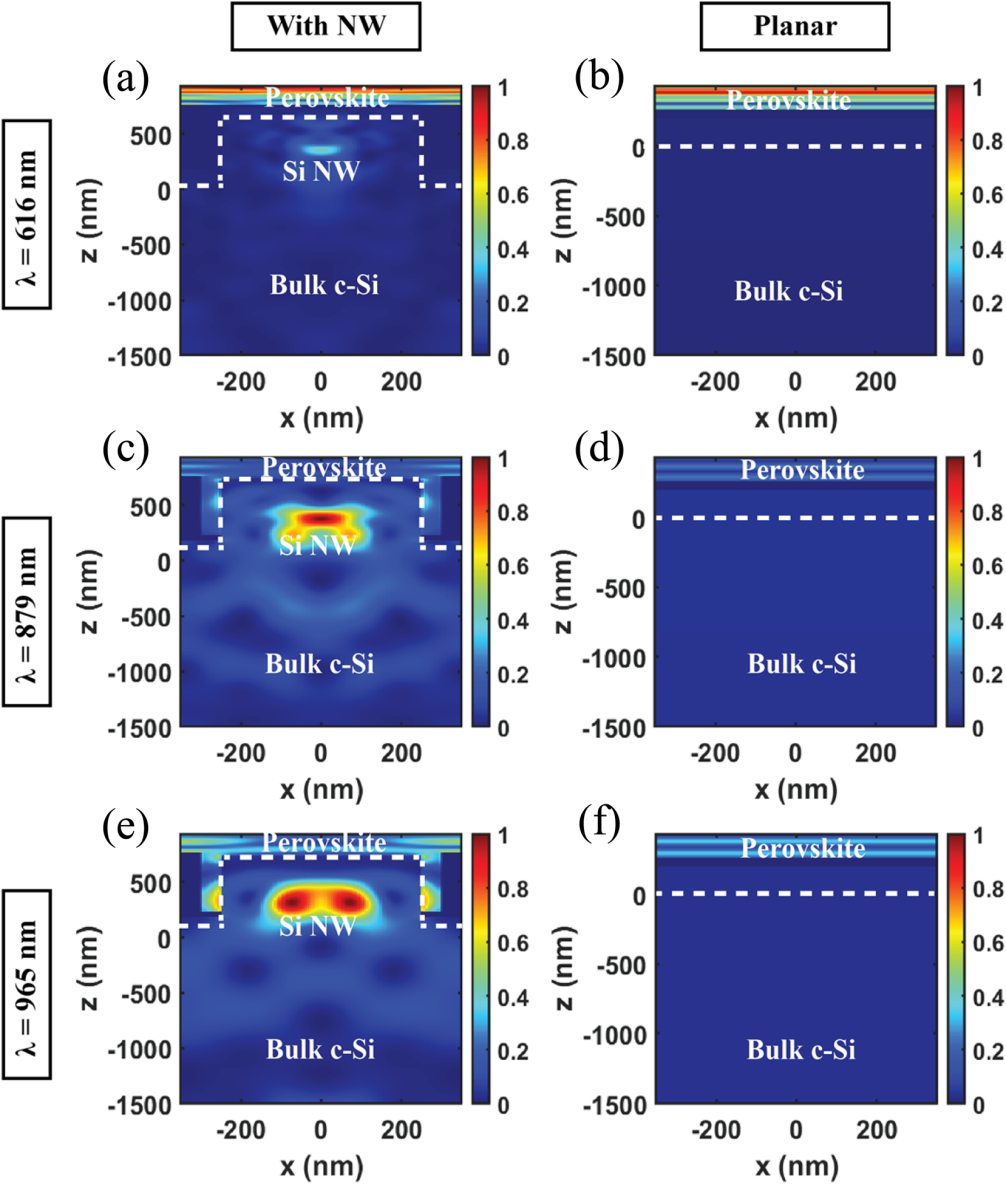
Normalized magnitude of the power absorption at *λ* = 616 nm, 879 nm, 965 nm for perovskite/Si NW tandem solar cell [(a), (c) and (e)] and perovskite/Si tandem cell [(b), (d) and (f)].

To elucidate the near-field coupling of incident light into the underlying c-Si absorber, the spatial distributions of the absorbed power density in the *z*–*x* plane, extracted from power monitors at *λ* = 616 nm, 879 nm, and 965 nm, are presented in Fig. 5(a), (c), and (e) for the Si NWs on c-Si. The corresponding absorption distributions for the planar c-Si reference are shown in Fig. 5(b), (d), and (f), respectively. At each wavelength, the transmitted power through the monitors is normalized to the maximum absorption obtained across both configurations. At shorter wavelengths (*e.g.*, 616 nm), where the photon energy exceeds the perovskite bandgap, absorption predominantly occurs within the perovskite top cell, leading to negligible coupling into the bottom c-Si layer. In contrast, at longer wavelengths (879 nm and 965 nm), which lie below the perovskite absorption edge, the incident light couples strongly into the Si NW and the underlying c-Si. Hence, for the NW configuration, pronounced absorption occurs not only within the Si NWs themselves but also in the underlying bulk, facilitated by enhanced scattering and redistribution of the incident field. This effect yields a substantially higher absorption compared to planar c-Si, further highlighting the role of NW geometry in facilitating light harvesting. Importantly, for planar c-Si, the simulations confirm negligible near-field coupling, with the incident light largely transmitted or reflected, resulting in minimal electric field penetration into the c-Si substrate. By contrast, the Si NW array introduces strong localized field enhancements and act as scattering centers that redirect and couple the incident light into the bottom Si subcell.

### 4.2 Fabry–Pérot resonance

**Fig. 6:**
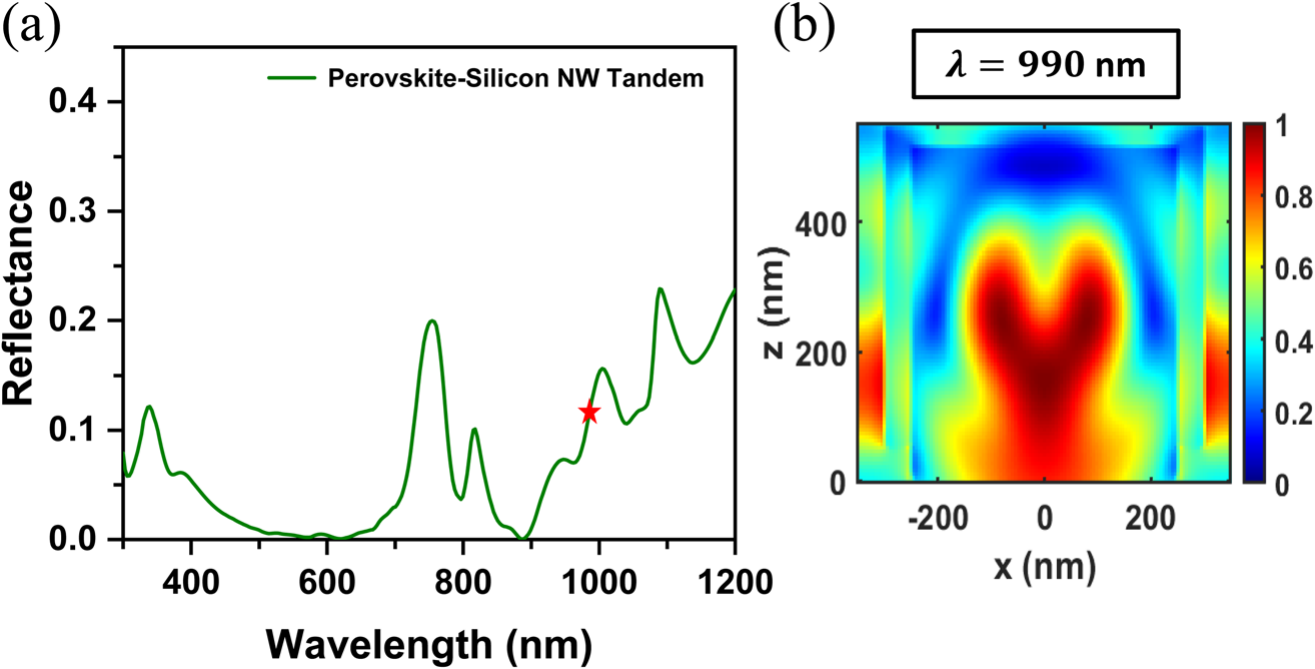
(a) Reflectance spectrum of the perovskite/Si NW tandem showing oscillations resulting from Fabry–Pérot resonances in the NW array (b) corresponding normalized magnitude of the electric field surrounding the NW at *λ* = 990 nm (marked with an asterisk in (a)).

Vertically aligned Si NWs, when illuminated along their long axis under normal incidence, can sustain Fabry–Pérot resonance modes. This effect is also observed in our proposed structure, as illustrated by the electric field distribution at *λ* = 990 nm in Fig. 6(b), confirming the presence of standing wave patterns indicative of Fabry–Pérot resonances. The nodes of the electric field are seen to be evenly spaced along the NW long-axis. Fabry–Pérot resonance occurs when the incident light reflects off both the top and bottom of the NW array, creating a standing wave that results in a distinct dip in the reflectance spectrum, as shown in Fig. 6(a). Formation of Fabry–Pérot cavities require the roundtrip NW length (2h) to be an integer (*m*) multiple of the effective wavelength (*λ*_eff_) of the NW guided mode such that 2h = *m* × *λ*_eff_, where *λ*_eff_ = *λ*_o_/*n*_eff_ with *λ*_o_ as the effective wavelength in the NW and *n*_eff_ as the effective refractive index. For our proposed NW array, this condition is fulfilled at multiple wavelengths, giving rise to oscillations in the reflectance spectrum of Fig. 6(a). Provided that the NWs are closely packed, Fabry–Pérot resonance can confine light in the gaps between the NWs.^34,40^ This can couple incident light into the bulk substrate as the substrate refractive index decreases. Given that the refractive index of Si decreases with increasing wavelength above the bandgap, the characteristic dips in the reflection spectrum also increases with increasing wavelength.^36^ The contribution of these effects to the overall absorption enhancement can be further evaluated by calculating the ratio of absorption within NWs to bulk Si absorption at specific wavelengths (Fig. S3, SI).

### 4.3 Optical carrier generation rate

**Fig. 7:**
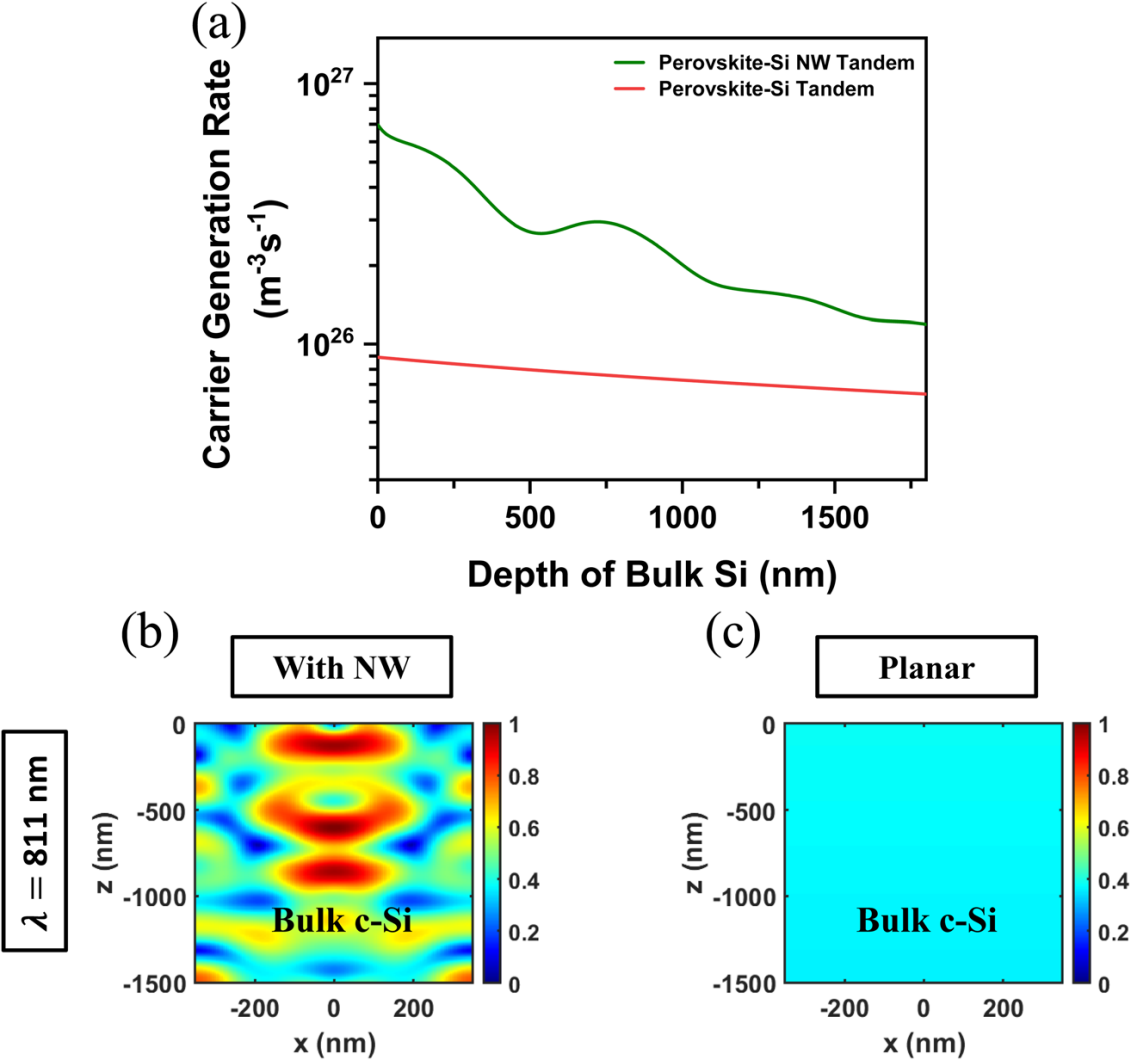
(a) Carrier generation rate within bulk c-Si as a function of Si depth for both perovskite/Si tandem and perovskite/Si NW tandem solar cells. Electric-field profile inside bulk c-Si at *λ* = 811 nm (b) with Si NW array on top (c) without Si NW array.

Fig. 7(a) illustrates the carrier generation rate inside the bulk c-Si layer, as a function of the c-Si thickness, with and without the NWs on top. With the Si NWs, on top of the c-Si, the tandem cell exhibits a significantly higher generation rate throughout the depth of bottom Si, particularly near the surface, due to improved light trapping and scattering. The contour plots at *λ* = 811 nm illustrate the electric field profiles inside the c-Si with (Fig. 7(b)) and without the NWs (Fig. 7(c)). The incorporation of NWs results in alternating bright and dark regions, indicating localized field enhancements, which are absent in the planar configuration. The enhanced carrier generation rate and oscillatory pattern in perovskite/Si tandem has been explained in previous studies, by taking into account, the lensing effect of the NWs.^41,42^ Lu, *et. al.*^41^ reported that NWs, due to their high aspect ratio and waveguiding ability, can form focused dark and bright spots, leading to enhanced photon absorption and carrier generation in certain regions in the bulk.

### 4.4 Tandem solar cell output characteristics

**Fig. 8:**
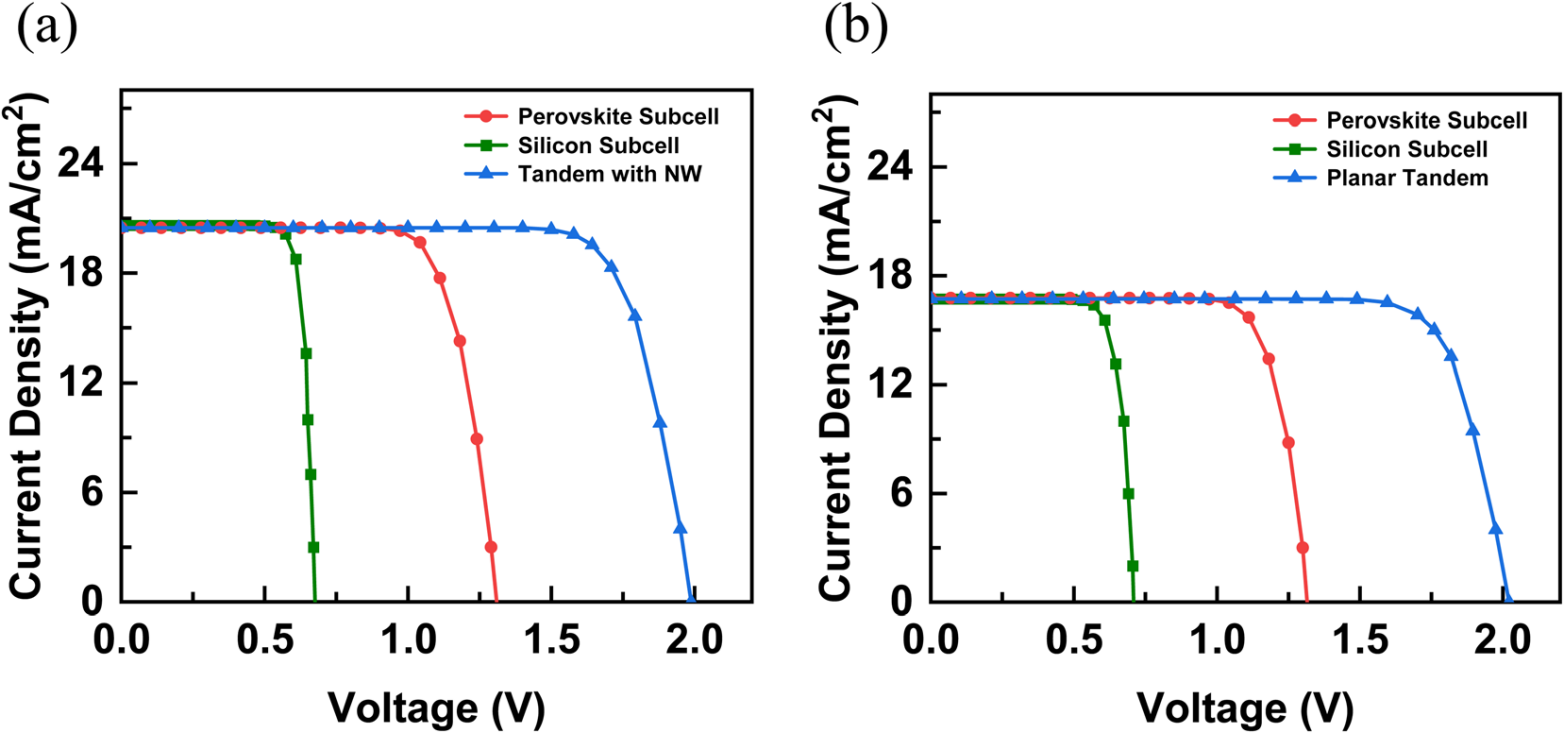
Calculated *J*–*V* characteristics of the (a) perovskite/Si NW tandem, (b) perovskite/Si planar tandem displaying both the individual contributions from the top and bottom subcells as well as the total device performance.

The 3D carrier generation rates for both the top perovskite and the bottom Si cell are spatially averaged to form a 1D generation profile, which is used by SCAPS-1D to extract the electrical characteristics of the tandem solar cell. The standalone top and bottom subcells are simulated separately and then for the tandem configuration, the subcell currents are matched by adjusting the thickness of the top perovskite as shown in Fig. 8(a) and (b). For c-Si without the NWs, current matching occurs when the thickness of the perovskite is 220 nm. When an array of Si NWs is grown on top, a thicker perovskite (∼530 nm) is needed to match the subcell currents. The bulk c-Si thickness is maintained at 180 µm in both configurations. The ITO–Si interface defect density was set to practical values for both planar and Si NW tandem configurations, with 10^15^ cm^−3^ for the Si NW case and 10^11^ cm^−3^ for the planar cell, reflecting the significantly smaller interface area in the latter.^43^ Consequently, the higher interface defect density in the Si NW structure resulted in a reduction in *V*_OC_ relative to its planar counterpart, which can be attributed to enhanced carrier recombination arising from the significantly larger ITO–Si contact area in the NW geometry. Table 1 summarizes the output characteristics of both the current matched tandem solar cells, with and without the NWs. The short-circuit current density (*J*_SC_) and PCE of the tandem cell, with the Si NW array on top, are 22.6% and 5.13% higher, respectively, than the corresponding values without the Si NWs. This improvement reflects the nanophotonic effects of the NW architecture, which enhance light absorption and consequently increase electron–hole pair generation within the device.

**Table 1:** Output characteristics of the 2-T tandem solar cells

Solar cell	*J*_SC_ (mA cm^−2^)	*V*_OC_ (V)	FF (%)	PCE (%)
2-T Perovskite/Si tandem	16.71	2.02	79.91	26.98
2-T Perovskite/Si NW tandem	20.48	1.99	78.79	32.11

**Fig. 9:**
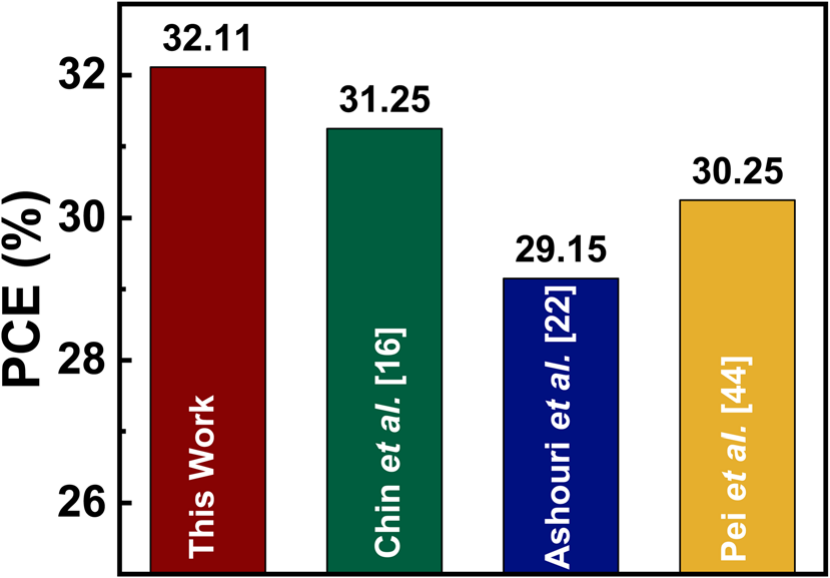
Comparison between the PCEs of the proposed design with state-of-the-art perovskite/Si tandems reported in literature.

To benchmark the performance of our proposed design within the current state of the art, we compared the PCE of our device with leading experimentally demonstrated perovskite-Si monolithic tandems reported in the literature. For instance, Chin *et al.* achieved a certified 31.25% efficiency through advanced interface passivation engineering.^16^ Ashouri *et al.* reported a 29.15% device enabled by reduced nonradiative recombination at the hole-selective interface,^22^ while Pei *et al.* demonstrated a 30.52% tandem using reactive passivation at the perovskite–ETL interface.^44^ In comparison, our Si NW-integrated tandem architecture attains a simulated PCE of 32.11%, exceeding the performance of these experimentally realized devices. This comparison, summarized in Fig. 9, underscores the strong potential of incorporating a NW-enhanced Si bottom subcell for next-generation monolithic perovskite/Si tandem photovoltaics.

## 5. Conclusion

In this work, we have proposed a novel 2-T perovskite/c-Si tandem solar cell, where an array of vertically aligned Si NWs is grown on top of the underlying c-Si. The Si NWs, which extend into the interlayer, connects the two subcells and significantly enhances the coupling of incident light from the top perovskite subcell to the bottom c-Si subcell through waveguiding effect and Fabry–Pérot resonance modes. Under current matched conditions, the proposed perovskite/Si tandem has 5.13% higher PCE than conventional perovskite/Si tandem solar cells without the NW array. The PCE can be further enhanced by optimizing the NW material, shape and geometry. This work presents a new insight towards minimizing interlayer losses and achieving highly efficient perovskite/Si tandems.

## Author contributions

Conceptualization: T. Rahman and M. Hossain; data curation, formal analysis, investigation, methodology, visualization, writing – original draft: A. Saha and M. A. Fatin; writing – review and editing: T. Rahman and M. Hossain; project administration and supervision: M. Hossain.

## Conflicts of interest

The authors declare no conflicts of interest.

## Supplementary Material

RA-015-D5RA07413D-s001

## Data Availability

All data included in this study are available from the corresponding authors upon request. For any specific inquiries, please contact Mainul Hossain E-mail: mainul.eee@du.ac.bd. Supplementary information (SI): Section S1: Calibration of the simulated top and bottom subcells. Section S2: Optimization of the Si NW geometry for maximum absorption. Section S3: Overall absorption enhancement. Fig S1: Calibrated *J*–*V* curves for the standalone subcells under AM1.5G incident light. Fig S2: Results of FDTD simulations with TM polarized incident light showing absorbance spectra (a–c) for different NW radii (*r*_NW_ = 90 nm, 170 nm, 250 nm) when periodicity (*P*) is varied, and (*d*) for different periodicities (*P* = 600 nm, 700 nm, 800 nm) at the optimum radius (*r*_NW_ = 250 nm). Table S1: Output parameters of the simulated subcells compared with experimental values. Table S2: Input parameters for simulated perovskite top subcell. Table S3: Input parameters for simulated Si bottom subcell. Table S4: Absorption ratio of Si NWs to bulk Si at selected wavelengths. See DOI: https://doi.org/10.1039/d5ra07413d.
