# Supplementary material for: Light trapping and guided mode enhancement in perovskite/Si tandem solar cells with embedded silicon nanowires
Source: RSC Adv. 2025 Dec 9;15(57):49063–73. doi: 10.1039/d5ra07413d (PMC12687930; doi:10.1039/d5ra07413d)
Supplement: RA-015-D5RA07413D-s001 [file RA-015-D5RA07413D-s001.pdf]

Supplementary Information (SI) for RSC Advances.
This journal is © The Royal Society of Chemistry 2025

Supplementary Information

Light trapping and guided mode enhancement in perovskite/Si tandem solar cells with embedded silicon nanowires

Arpan Saha,[1,†] Mohammad Ajmain Fatin,[1,†] Tasmiat Rahman,[2] and Mainul Hossain[1,*]

[1]*Department of Electrical and Electronic Engineering, University of Dhaka, Dhaka-1000, Bangladesh*
[2]*Department of Electronics and Computer Science, University of Southampton, Southampton SO17 1BJ, United Kingdom.*
**mainul.eee@du.ac.bd*
[†]*These authors contributed equally.*

S1. Calibration of the simulated top and bottom subcells

To ensure that the optical and electrical models of the proposed tandem cell represent realistic device behavior, we calibrated both the top ($Cs_{0.18}FA_{0.82}Pb(I,Br)_3$) and the bottom (c-Si) subcells against experimental data from Chin *et al.* [1]. The calibrated *J–V* characteristics of the subcells, presented in Fig. S1, demonstrate a close agreement with the experimental measurements and establish a reliable baseline prior to introducing the nanowire integrated interlayer. Table S1 compares the simulated and the experimental output parameters.

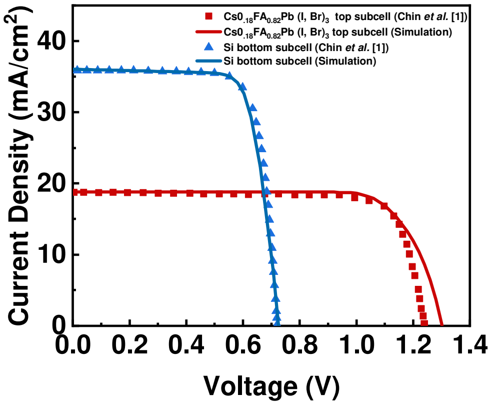

Fig. S1. Calibrated *J-V* curves for the standalone subcells under AM1.5G incident light. Symbols represent the experimental results reported by Chin *et al.* [1] and the solid lines represent the simulated data.

Table S1. Output parameters of the simulated subcells compared with experimental values by Chin *et al.* [1]

Solar Cell	J_{SC} (mA/cm^2)	V_{OC} (V)	FF (%)	PCE (%)
Top Perovskite (Experiment by Chin *et al.*)	18.90	1.24	78.80	18.46
Top Perovskite (Simulation)	18.81	1.30	77.63	19.00
Bottom c-Si (Experiment by Chin *et al.*)	35.85	0.72	77.53	20.00
Bottom c-Si (Simulation)	36.12	0.73	75.72	19.82

The input parameters used in simulating the *J-V* characteristics of the perovskite and the Si subcells, are listed below in Table S2 and S3, respectively.

Table S2. Input parameters for simulated perovskite top subcell

Parameter	SnO_2	C_{60}	$Cs_{0.18}FA_{0.82}Pb(I,Br)_3$	Me-4PACz
Thickness (nm)	10*	20*	220-530*	5*
N_a (cm^{-3})	0	0	1.00×10^{10}	1.00×10^{17}
N_d (cm^{-3})	1.00×10^{19}	1.00×10^{19}	0	0
ε_s	8	5	10	4
χ (eV)	3.9	4	3.87	2.8
E_g (eV)	3.6	2.2	1.71	3.3
N_C (cm^{-3})	3.16×10^{18}	2.2×10^{18}	2.76×10^{18}	1×10^{19}
N_V (cm^{-3})	2.5×10^{19}	1.8×10^{19}	3.9×10^{18}	1×10^{19}
μ_n (cm^2/ V.s)	15.0	.01	5	.001
μ_p (cm^2/ V.s)	0.1	.01	5	5
N_{def}	1.00×10^{15}	1.00×10^{15}	1.00×10^{15}	1.00×10^{15}
References	[2]	[2]	[2]	[2]

Table S3. Input parameters for simulated Si bottom subcell

Parameter	n+ c-Si	p c-Si	p+ c-Si	ITO
Thickness (nm)	500*	169500*	10000*	50*
N_a (cm^{-3})	0	1.00×10^{16}	1.00×10^{20}	0
N_d (cm^{-3})	1.00×10^{20}	0	0	1.00×10^{21}
ε_s	11.9	11.9	11.9	9
χ (eV)	4.05	4.05	4.05	4
E_g (eV)	1.12	1.12	1.12	3.5
N_C (cm^{-3})	2.819×10^{19}	2.819×10^{19}	2.819×10^{19}	2.20×10^{18}
N_V (cm^{-3})	1.04×10^{19}	1.04×10^{19}	1.04×10^{19}	1.80×10^{18}
μ_n (cm^2/ V.s)	1400	1400	1400	20
μ_p (cm^2/ V.s)	450	450	450	10
N_{def}	1.00×10^{15}	1.00×10^{15}	1.00×10^{15}	1.00×10^{15}
References	[3]	[3]	[3]	[4]

S2. Optimization of the Si NW geometry for maximum absorption

To remain consistent with experimentally stable and lithography-compatible structures, we selected a NW height of 500 nm, which provides strong optical coupling while preserving the structural robustness in the proposed monolithic tandem stack. For this reason, significantly taller nanowire geometries were not pursued. The geometric optimization performed in this work—including variations in nanowire radius and periodicity—is presented in the Fig. S2. The optimal radius and period were identified as 250 nm and 700 nm, respectively, offering broadband absorption enhancement across the simulated spectral range.

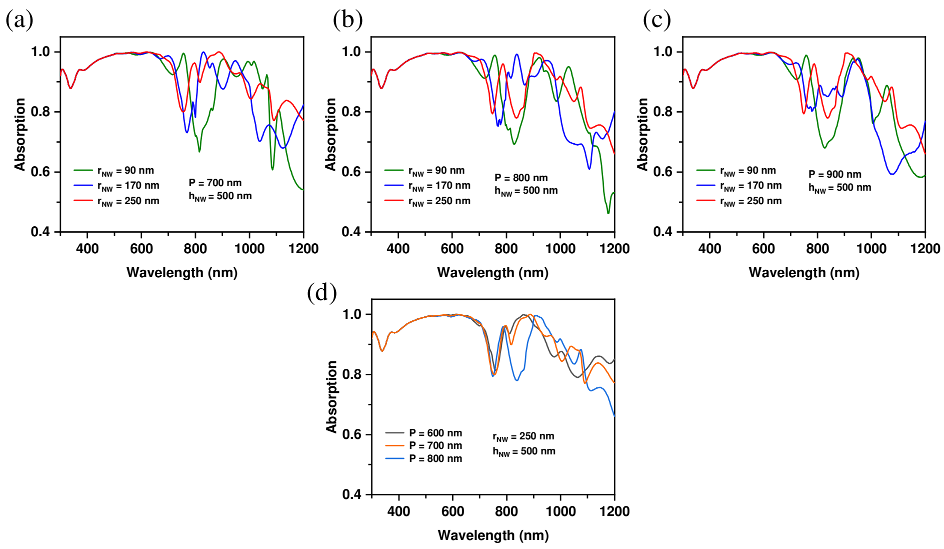

Fig. S2. Results of FDTD simulations with TM polarized incident light showing absorbance spectra (a-c) for different NW radii (r_{NW} = 90 nm, 170 nm, 250 nm) when periodicity (P) is varied, and (d) for different periodicities (P = 600 nm, 700 nm, 800 nm) at the optimum radius (r_{NW} = 250 nm). Height of the NW was kept constant at 500 nm. The optimized radius (r_{NW}) and periodicity (P) are determined as 250 nm and 700 nm, respectively. The (n, k) data used in the FDTD simulations and the electrical parameters for the SCAPS-1D simulations have been directly extracted from Yang *et al*. [2]

S3. Overall absorption enhancement

To quantitatively evaluate the optical contribution of the nanowire (NW) geometry within the tandem device, we computed the absorption (A_{NW}) in the Si NWs and compared it with that of a planar bulk-Si layer (A_{Bulk}). Because the wide-bandgap perovskite top cell absorbs the majority of visible photons before they reach the c-Si bottom subcell, the Si NWs receive only the residual transmitted visible light and the near-infrared (NIR) photons that are not absorbed by the top cell. To capture this behavior, we evaluated the overall absorption enhance (AE) as:

$$AE(\lambda) = \frac{A_{NW}(\lambda)}{A_{Bulk}(\lambda)}$$

at four representative wavelengths: two in the residual visible range (493 nm and 613 nm) and two in the NIR region where most photons reach the Si bottom cell (716 nm and 881 nm). The results are summarized in Table 1 below:

Table S4. Absorption ratio of Si NWs to bulk Si at selected wavelengths

Wavelength (nm)	Si NW Absorption (A_{NW})	Si Bulk Absorption (A_{Bulk})	$AE(\lambda)$
493	0.026	0.006	4.333
613	0.030	0.123	0.244
716	0.041	0.351	0.117
881	0.018	0.917	0.020

Because the perovskite layer filters out nearly all visible wavelengths, the Si absorption between 493–613 nm is extremely low. The relatively large ratios in this range reflect the weak residual photon flux rather than meaningful photocurrent generation; although the NWs absorb slightly more of this remaining light than planar Si, its overall contribution to current is negligible. The near-infrared region (716–881 nm) is far more relevant, as it carries most of the photons that reach the bottom c-Si subcell. Here, the absorption ratios fall to 0.117 and 0.020, indicating that Si NWs absorb only 2–12% of the NIR light that planar Si would absorb. This confirms that the NW region functions primarily as a low-loss optical transmission layer: long-wavelength photons are guided or funneled through the NW architecture and delivered efficiently to the underlying c-Si. This quantitative analysis is fully consistent with the field-distribution and resonance behavior described in the manuscript. It demonstrates that the NW architecture does not enhance tandem performance by absorbing additional visible light—already harvested by the perovskite—but rather by minimizing NIR losses and enabling efficient photon delivery to the c-Si bottom cell.

References:

1. X. Y. Chin, D. Turkay, J. A. Steele, S. Tabean, S. Eswara, M. Mensi, P. Fiala, C. M. Wolff, A. Paracchino, K. Artuk, D. Jacobs, Q. Guesnay, F. Sahli, G. Andreatta, M. Boccard, Q. Jeangros, and C. Ballif, "Interface passivation for 31.25%-efficient perovskite/silicon tandem solar cells," Science **381**(6653), 59–63 (2023).
2. J. Yang and Q. Bao, "Enhancing perovskite-silicon tandem solar cells through numerical optical and electric optimizations for light management," Opt. Express **32**(6), 8614–622 (2024).
3. K. Kim, J. Gwak, S. K. Ahn, Y. J. Eo, J. H. Park, J. S. Cho, M. G. Kang, H. E. Song, and J. H. Yun, "Simulations of chalcopyrite/c-Si tandem cells using SCAPS-1D," Sol. Energy **145**, 52–58 (2017).
4. M. K. Hossain, M. H. K. Rubel, G. F. I. Toki, I. Alam, M. F. Rahman, and H. Bencherif, "Effect of Various Electron and Hole Transport Layers on the Performance of $CsPbI_3$-based perovskite solar cells: A numerical investigation in DFT, SCAPS-1D, and wxAMPS frameworks," ACS Omega **7**(47), 43210–43230 (2022).
